# Multiple roles of mitochondrial autophagy receptor FUNDC1 in mitochondrial events and kidney disease

**DOI:** 10.3389/fcell.2024.1453365

**Published:** 2024-10-09

**Authors:** Kaiqing Li, Xue Xia, Ying Tong

**Affiliations:** ^1^ Heilongjiang University of Chinese Medicine, Harbin, China; ^2^ The First Affiliated Hospital of Heilongjiang University of Chinese Medicine, Harbin, China

**Keywords:** FUNDC1, mitochondrial autophagy, kidney disease, phosphorylation, dephosphorylation

## Abstract

This article reviews the latest research progress on the role of mitochondrial autophagy receptor FUN14 domain containing 1 (FUNDC1) in mitochondrial events and kidney disease. FUNDC1 is a protein located in the outer membrane of mitochondria, which maintains the function and quality of mitochondria by regulating mitochondrial autophagy, that is, the selective degradation process of mitochondria. The structural characteristics of FUNDC1 enable it to respond to intracellular signal changes and regulate the activity of mitochondrial autophagy through phosphorylation and dephosphorylation. During phosphorylation, unc-51-like kinase 1 (ULK1) promotes the activation of mitophagy by phosphorylating Ser17 of FUNDC1. In contrast, Src and CK2 kinases inhibit the interaction between FUNDC1 and LC3 by phosphorylating Tyr18 and Ser13, thereby inhibiting mitophagy. During dephosphorylation, PGAM5 phosphatase enhances the interaction between FUNDC1 and LC3 by dephosphorylating Ser13, thereby activating mitophagy. BCL2L1 inhibits the activity of PGAM5 by interacting with PGAM5, thereby preventing the dephosphorylation of FUNDC1 and inhibiting mitophagy. FUNDC1 plays an important role in mitochondrial events, participating in mitochondrial fission, maintaining the homeostasis of iron and proteins in mitochondrial matrix, and mediating crosstalk between mitochondria, endoplasmic reticulum and lysosomes, which have important effects on cell energy metabolism and programmed death. In the aspect of kidney disease, the abnormal function of FUNDC1 is closely related to the occurrence and development of many diseases. In acute kidney injury (AKI), cardiorenal syndrome (CRS), diabetic nephropathy (DN), chronic kidney disease (CKD) ,renal fibrosis (RF) and renal anemia, FUNDC1-mediated imbalance of mitophagy may be one of the key factors in disease progression. Therefore, in-depth study of the regulatory mechanism and function of FUNDC1 is of great significance for understanding the pathogenesis of renal disease and developing new treatment strategies.

## 1 Introduction

Mitochondria are intracellular signal organelles that provide ATP to cells through oxidative phosphorylation (Kim and Lee, 2024). They are also the main sites for fatty acid oxidation and reactive oxygen species (ROS) formation (Song Y. et al., 2024). When mitochondria are dysfunctional or damaged, iron homeostasis may be affected and oxidative stress may be induced, increasing the risk of lipid accumulation (Fang et al., 2022). In addition, iron overload in mitochondria may cause Fenton response, leading to the production of ROS, and the leakage of these ROS from mitochondria may lead to cell damage and inflammation (Takahashi et al., 2021). Therefore, excessive ROS formation may further damage mitochondria and lead to their dysfunction. In order to maintain cell stability and health, timely removal of these dysfunctional mitochondria is very important.

**FIGURE 1:**
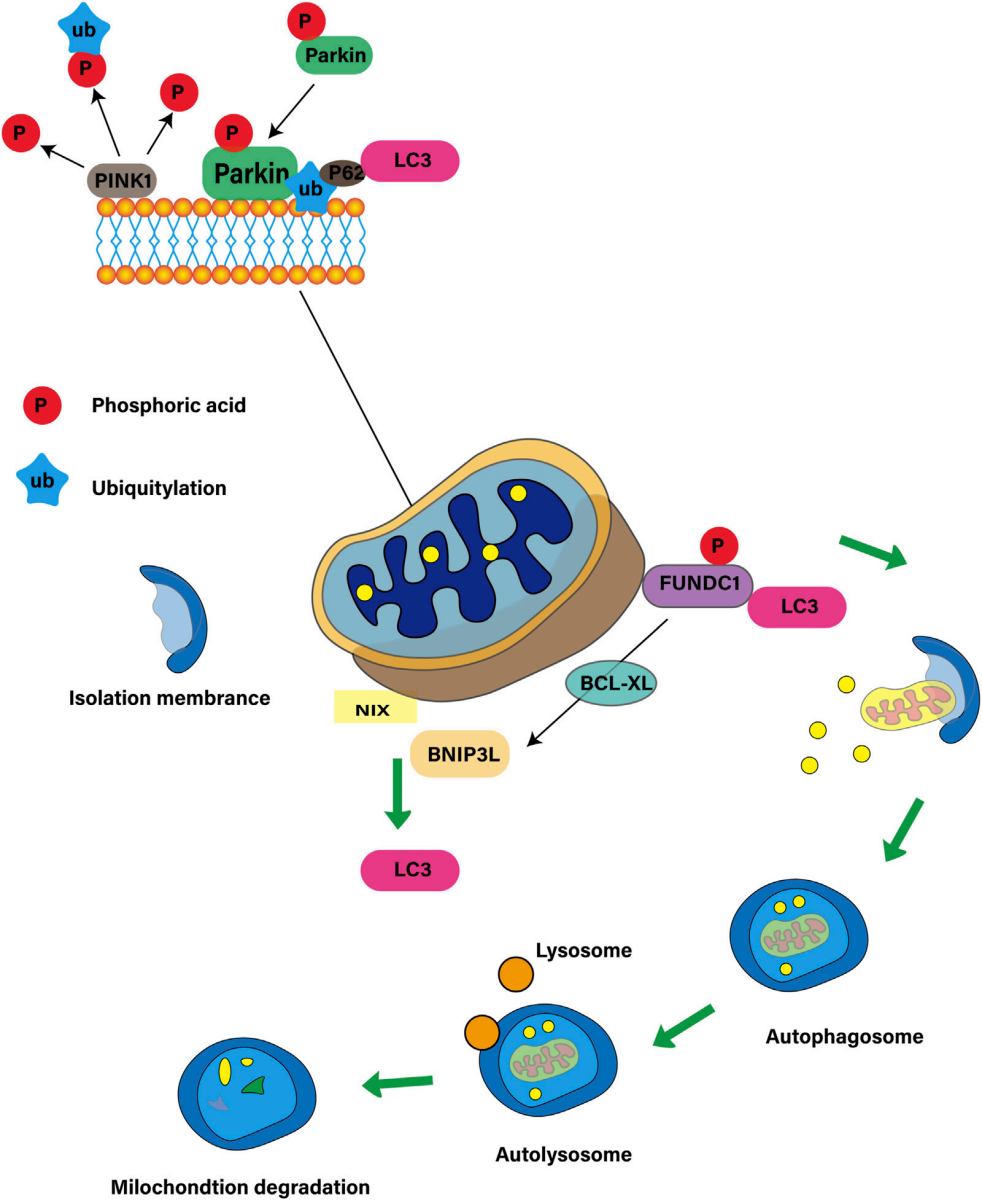
Mitochondrial autophagy ubiquitin-dependent and non-ubiquitin-dependent pathways.

Mitochondrial autophagy is a selective autophagy process that maintains the balance between the number and function of mitochondria in cells by identifying and removing damaged or redundant mitochondria (Degli Esposti, 2024). This process is essential for cell function, metabolic regulation and stress response, involving both ubiquitin-dependent and non-ubiquitin-dependent pathways. The ubiquitin-dependent pathway centers on PINK1 and Parkin. PINK1 locates on the depolarized mitochondria and activates Parkin when the membrane potential is damaged, triggering autophagy (Hu et al., 2024). The non-ubiquitin-dependent pathway is dominated by mitochondrial autophagy receptors such as BNIP3 (Kim et al., 2021), NIX/BNIP3L (Nguyen-Dien et al., 2023), FUNDC1 (Chen D. et al., 2024) and PHB2 (Liu et al., 2024), which contain LC3 interactive motifs (LIR), which directly bind to autophagy-related proteins, initiate autophagy, regulate the quantity and quality of mitochondria, maintain energy balance and resist cell damage (Terešak et al., 2022). As shown in Figure 1.

As a new type of mitochondrial receptor protein in mammalian cells, FUNDC1 can mediate mitochondrial autophagy under hypoxia (Deng et al., 2024). As an important metabolic organ of human body, kidney depends on a large amount of ATP produced by tricarboxylic acid cycle to meet its high energy needs. Mitochondrial dysfunction may lead to a variety of kidney diseases (Govers et al., 2021). Recent studies have revealed the close relationship between FUNDC1 and kidney disease, and pointed out that mitochondrial dysfunction can be improved by regulating the activity of FUNDC1, which provides a new molecular target for the treatment of kidney disease (Wu et al., 2022). This article will review the multifaceted role of mitochondrial autophagy receptor FUNDC1 in mitochondrial events and renal diseases, in order to provide a theoretical basis and new perspective for the research and treatment of renal diseases.

## 2 Structure and function of mitochondrial autophagy receptor FUNDC1

**FIGURE 2:**
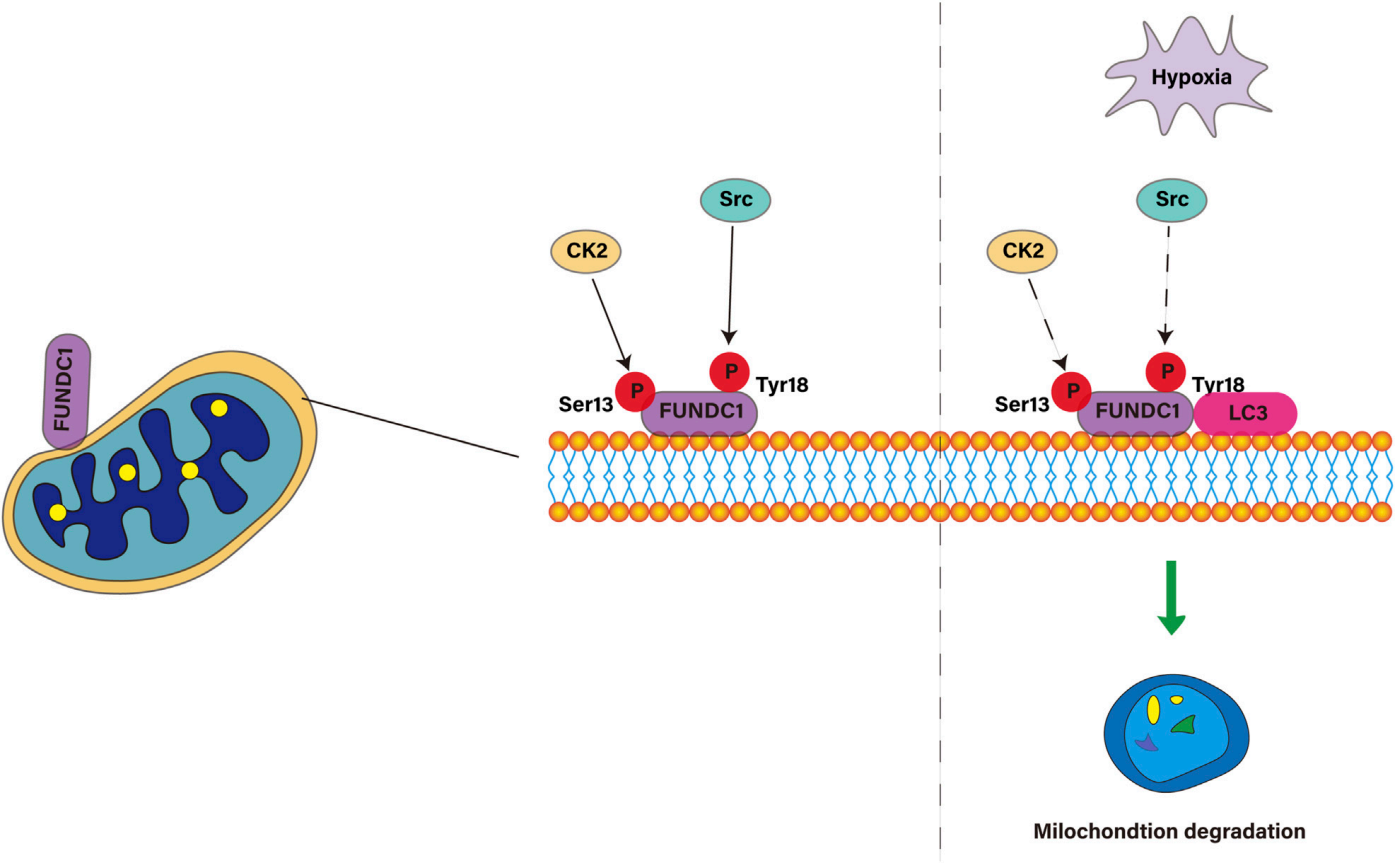
Molecular mechanism of FUNDC1 regulating mitophagy under adequate and hypoxic conditions.

FUNDC1 is a key protein located in the outer membrane of mitochondria, which regulates mitochondrial autophagy through changes in phosphorylation state. FUNDC1 consists of 155amino acids and has three α-helical transmembrane domains. The N-terminal of FUNDC1 is exposed in the cytoplasm and the C-terminal is located in the gap between the inner and outer membrane of mitochondria. The N-terminal of FUNDC1 contains a LC3 interaction region LIR with a motif of (Y18-E19-V20-L21). Under the normal condition of sufficient oxygen, the Tyr18 and Ser13 sites in the LIR motif of FUNDC1 are phosphorylated by Src and CSNK2/CK2 kinase, respectively, which leads to the inactive state of FUNDC1, which inhibits the binding with LC3, thus preventing mitochondrial autophagy. However, under hypoxic conditions, the inactivation of Src and CK2 kinases and the dephosphorylation of FUNDC1 enable them to bind to LC3 and induce mitochondrial autophagy. Overexpression of FUNDC1 increased mitochondrial autophagy and cell proliferation, while knocking down the expression of FUNDC1 inhibited mitochondrial autophagy and cell proliferation induced by hypoxia (Liu et al., 2022), which is consistent with the results of Pan and colleagues (Pan et al., 2021). In this process, the phosphorylation state of Tyr18 acts as a molecular switch to regulate the interaction between FUNDC1 and LC3 (Kuang et al., 2016). The binding mechanism induced by dephosphorylation of FUNDC1 is contrary to that of other proteins containing LIR motifs, whose phosphorylation usually increases the affinity for LC3 binding. In general, FUNDC1 plays an important role in cell response to hypoxic stress through the interaction between LIR and LC3. Its highly conservative characteristics and high expression in a variety of tissues make it a valuable molecular target for the study of mitochondrial function and related diseases, especially kidney diseases. As shown in Figure 2.

## 3 Proteins involved in FUNDC1-mediated mitochondrial autophagy

FUNDC1-mediated mitochondrial autophagy is an important intracellular quality control mechanism, which involves the interaction of a variety of proteins. In this process, the phosphorylation and dephosphorylation of FUNDC1 play a key role in its activity, coordinate the regulation of mitochondrial autophagy and maintain the stability of the intracellular environment.

### 3.1 Phosphorylation of FUNDC1 inhibits mitophagy

#### 3.1.1 ULK1

ULK1 is activated under hypoxia or under the use of mitochondrial uncoupling agent and transferred to damaged mitochondria (Wu W. et al., 2014). It interacts with FUNDC1 protein and phosphorylates at the Ser17 site of FUNDC1, thus enhancing the binding of FUNDC1 to LC3, which is necessary for mitochondrial autophagy (Zhu et al., 2022). If the ULK1 binding site of FUNDC1 is mutated, it will prevent the transfer of ULK1 and the progress of mitochondrial autophagy. However, even in ULK1-disabled cells, mitochondrial autophagy can be restored by using ULK1 with kinase activity and phosphorylated mimic FUNDC1 mutants (Torii and Shimizu, 2020). This suggests that ULK1 regulates its recruitment to damaged mitochondria by phosphorylating FUNDC1, which is essential for mitochondrial autophagy. ULK1 complex, composed of ULK1 or ULK2, FIP200 and mATG13, is a bridge between upstream nutritional or energy receptors mTOR (Deng et al., 2023) and AMPK (Cai et al., 2022a) and downstream autophagosomes. Over-activation of AMPK α 1/ULK1/FUNDC1/mitochondrial autophagy pathway maintains mitochondrial function (Yang et al., 2023), normalizes mitochondrial fission and fusion, neutralizes the concentration of hyperphysiological reactive oxygen species and inhibits mitochondrial apoptosis (Cai et al., 2022a). Under the condition of starvation or hypoxia, AMPK is activated and mTOR is inactivated, which promotes the phosphorylation of Ser317 (Wang W. et al., 2022), Ser467, Ser555, Ser574 and Ser637 (Wang L. et al., 2022) sites in ULK1, thus promoting autophagy, while in the case of adequate nutrition, AMPK is inactivated, and the binding of mTOR with ULK1’s Ser757 (Han et al., 2021) site inhibits ULK1-AMPK interaction, resulting in ULK1 inactivation and autophagy signal closure. Therefore, the interaction between ULK1 and FUNDC1 and its subsequent phosphorylation events play a central role in the occurrence and regulation of mitochondrial autophagy.

#### 3.1.2 Protein kinases Src and CK2

Src and CK2 are two protein kinases that play key roles in cell growth, differentiation, proliferation and survival. Src kinase is a tyrosine kinase, which is upregulated by autophosphorylation at Y416 site and decreased by phosphorylation at Y507 site (Kim et al., 2020). Src inhibits mitochondrial autophagy (Chu et al., 2023) mediated by FUNDC1, a protein located in the outer membrane of mitochondria, by phosphorylating Tyr18 sites under physiological conditions.The study found that the activation of Src is associated with the inactivation of FUNDC1 (Tang et al., 2023), which is consistent with the original findings of Liu L et al. (Liu et al., 2012). On the other hand, CK2 is a constitutive serine/threonine kinase (Kim and Koh, 2023), which is related to the phosphorylation of FUNDC1 at the Ser13 site and was initially described as an inhibitor of FUNDC1 (Chen et al., 2014). It has been found that the inhibition of FUNDC1-related mitochondrial autophagy is related to the mitochondrial homeostasis interfered by CK2 (Zhou et al., 2018). Under normal conditions, the activation of Src and CK2 kinases leads to phosphorylation of Tyr18 and Ser13 sites of FUNDC1, which inhibits mitochondrial autophagy. This is because the phosphorylated FUNDC1 may conflict with the hydrophobic sac of LC3 and reduce its binding affinity to LC3. However, under long-term hypoxia, the inactivation of Src and CK2 kinases leads to dephosphorylation of FUNDC1 at Tyr18 and Ser13 sites, which promotes mitochondrial autophagy. Dephosphorylated FUNDC1 weakens the spatial interference of interaction with LC3 through conformational modification, resulting in co-localization of FUNDC1 and LC3-II, thus promoting mitochondrial autophagy.

### 3.2 Dephosphorylation of FUNDC1 activates mitophagy

#### 3.2.1 PGAM5

PGAM5 in mitochondria is a key Ser/Thr phosphatase, which participates in the regulation of mitochondrial autophagy and mitochondrial unfolded protein response, and plays a vital role in maintaining mitochondrial functional balance (Cai et al., 2023). When the mitochondrial membrane potential decreases, PGAM5 is activated, which works with PINK1 kinase to promote the transfer of PINK1 from intima to outer membrane and bind to Parkin, initiating the process of mitochondrial autophagy (Sekine et al., 2012). At the same time, some studies have suggested that PGAM5 helps to insert vacuolar cytotoxin An into the mitochondrial inner membrane to destroy the membrane potential, thus inducing mitochondrial autophagy (Wang L. et al., 2022). But the intermediate link is always the same. PGAM5 activates FUNDC1 by dephosphorylating the Ser13 site of FUNDC1, which enhances the binding ability of FUNDC1 and LC3 and promotes the interaction between them, which leads to the formation of selective autophagy and the clearance of damaged mitochondria (Ye et al., 2023). At the same time, CK2 can reverse the dephosphorylation of PGAM5 (Chen et al., 2014) by phosphorylating FUNDC1. Therefore, PGAM5 and CK2 jointly constructed a feedback mechanism to connect the stress response of mitochondria to the phosphorylation state of FUNDC1, and then control the regulation of mitochondrial autophagy.

#### 3.2.2 BCL2L1

**FIGURE 3:**
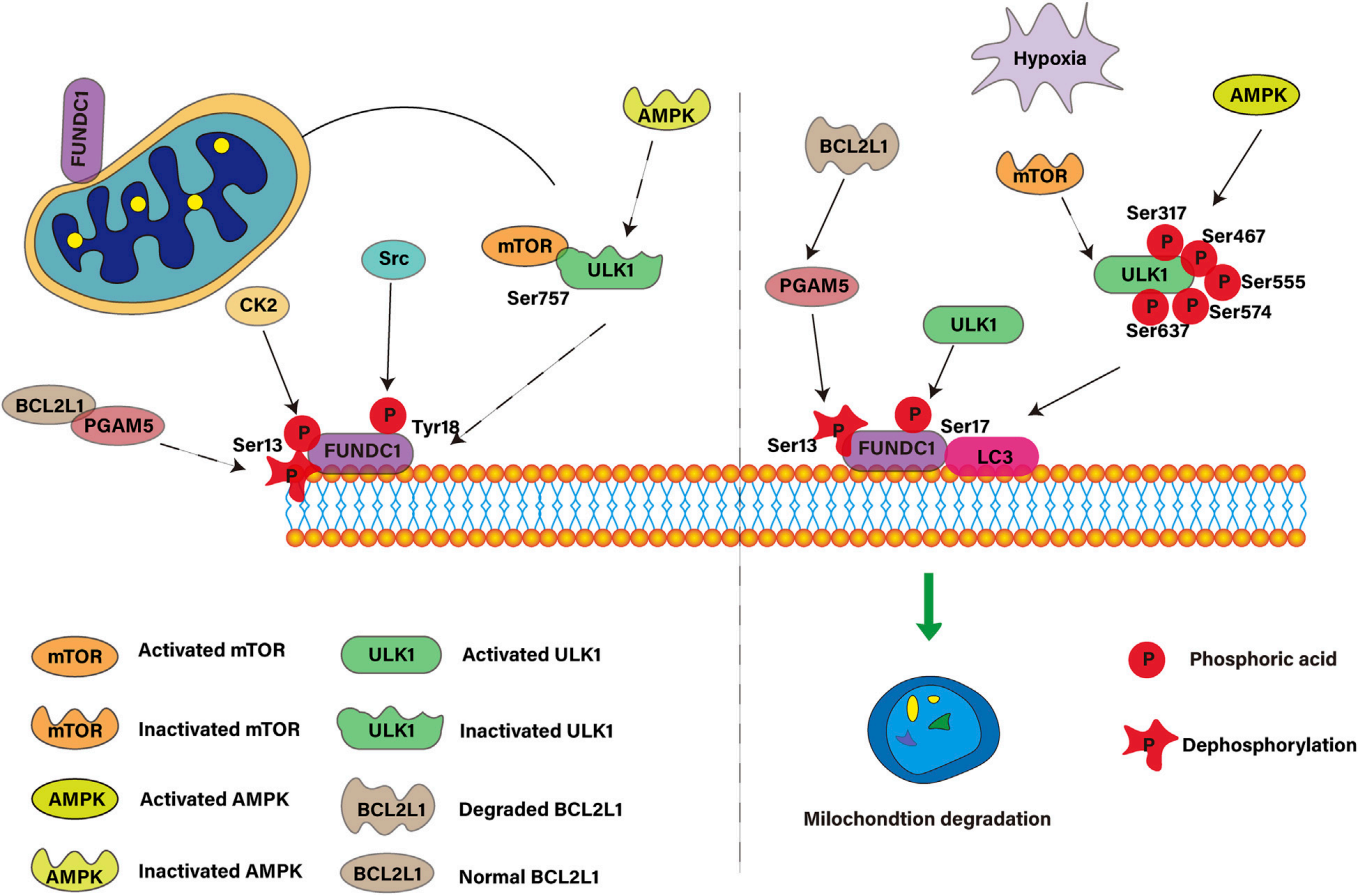
Proteins involved in FUNDC1-mediated mitophagy.

BCL2L1 is an important member of the Bcl-2 family and a key component of the mechanism of cell survival and death (Keller et al., 2023). Its main function is to inhibit apoptosis (Moriishi et al., 2023). Under the condition of normal oxygen content, BCL2L1 interacts with PGAM5 through its BH3 domain to inhibit the activation of PGAM5, thus preventing the dephosphorylation of FUNDC1 at the Ser13 site, thereby inhibiting mitochondrial autophagy. However, under hypoxic conditions, BCL2L1 will degrade, resulting in the release and activation of PGAM5. Activated PGAM5 promotes dephosphorylation of FUNDC1 at the Ser13 site. This dephosphorylated FUNDC1 interacts with LC3 and activates mitochondrial autophagy (Wu H. et al., 2014). Studies have shown that the expression level of BCL2L1 determines the level of PGAM5-mediated FUNDC1 dephosphorylation and mitochondrial autophagy (Ma et al., 2020). Even if the level of BCL2L1 remains the same, PGAM5 knockout can inhibit mitochondrial autophagy (Hashino et al., 2022). Therefore, the BCL2L1-PGAM5-FUNDC1 axis plays an important role in receptor-mediated mitochondrial autophagy under hypoxia. Under different pathophysiological conditions, how cells perceive external stimuli and regulate the dephosphorylation state of FUNDC1, and how PGAM5/BCL2L1 gene polymorphism affects this process, will be the focus of future research. As shown in Figure 3.

## 4 The role of mitochondrial autophagy receptor FUNDC1 in mitochondrial events

### 4.1 FUNDC1 is involved in mitochondrial fission

The health status and function of mitochondria are precisely regulated by their kinetic balance, including fission and fusion (Adebayo et al., 2021). Mitochondrial autophagy requires mitochondrial fission (Fukuda et al., 2023). Mitochondrial fission is a key process to maintain the health of mitochondria. It not only affects the metabolic state and survival of cells, but also plays a role in cytochrome C-mediated apoptosis (Yako et al., 2021). Dynamic protein-associated protein 1 (DRP1, also known as DNM1L) is recruited to the outer membrane of mitochondria and the existence of OPA1 in the inner membrane of mitochondria, which jointly regulates the balance of mitochondrial fission and fusion (Tsushima et al., 2018). Studies have shown that FUNDC1 coordinates mitochondrial fission and autophagy (Chen et al., 2016) by interacting with the K70 residues of DNM1L/DRP1 and OPA1. Under stress conditions, dephosphorylation of FUNDC1 promotes its dissociation from OPA1 (Ma et al., 2023) and binding with DRP1 (Xu A. et al., 2024), which mediates the “coupling” across double membranes and realizes the synergistic effect of mitochondrial dynamics and quality control. Under anoxic conditions, the membrane structure (MAM) associated with endoplasmic reticulum plays a key role in mitochondrial fission. FUNDC1 accumulates on MAMs through the interaction with calcitonin, and its binding to calnexin decreases with the progress of mitochondrial autophagy. This process promotes the recruitment of DRP1 and completes the mitochondrial fission (Wu et al., 2016). At the same time, USP19, a deubiquitin enzyme, is recruited to the endoplasmic reticulum under hypoxia, removes the ubiquitin chain from FUNDC1, stabilizes FUNDC1 in MAM, and assists in DRP1 recruitment (Chai et al., 2021). Although the interaction between FUNDC1 and calcitonin and its dissociation mechanism under hypoxia have not been fully elucidated, these processes are essential for the occurrence of mitochondrial fission and autophagy. Future studies need to explore how USP19 cooperates to regulate the ubiquitin state of FUNDC1 and the potential interaction between USP19 and calcitonin, which will contribute to a better understanding of the relationship between mitochondrial fission and autophagy.

### 4.2 FUNDC1 is involved in maintaining iron homeostasis in mitochondrial matrix

Mitochondrial autophagy is a key process in the regulation of iron metabolism and iron death. Under normal circumstances, mitochondrial autophagy releases iron ions by degrading ferritin, which are then transported to the mitochondria as a buffer mechanism. However, when the O-GlcNAcylation modification is reduced, the mitochondrial structure is damaged, resulting in more mitochondrial autophagy, which releases more iron ions and increases the sensitivity of cells to iron death (Yu et al., 2022). In addition, mitochondria are not only the main source of intracellular ROS, but also the accumulation of ROS can promote iron death (Fan et al., 2024) in high-speed rail environment. Iron loss will induce mitochondrial autophagy (Hara et al., 2020). In the mitochondrial matrix, FUNDC1 is involved in regulating the metabolism of iron ions in the mitochondrial matrix, which helps to maintain iron homeostasis. This process is very important to prevent excessive accumulation of iron ions in cells and to avoid oxidative stress and cell damage caused by it. SongK et al. found (Song K. et al., 2024) that extra ferrous ions are released after autophagy of mitochondria, which destroys iron homeostasis, which aggravates lipid peroxidation and eventually leads to iron death in cells. As the assembly and output center of iron-sulfur cluster (ISC), mitochondria play an important role in the regulation of intracellular iron metabolism. In pancreatic cancer, autophagy supports mitochondrial metabolism by regulating iron homeostasis, while autophagy inhibition reduces ISC formation by affecting ISC assembly protein 1 (Mukhopadhyay et al., 2023). Iron regulatory protein 1 (IRP1) plays a key role in mitochondrial phagocytosis induced by iron stress. IRP1 can inhibit the translation of Bcl-xLmRNA, while Bcl-xL is an inhibitory protein of mitochondrial phosphatase PGAM5, which can catalyze the dephosphorylation of FUNDC1 to activate mitochondria. WuH’s team found that disturbances in ISC biosynthesis inhibit Bcl-xL translation through the IRP1/Bcl-xL axis, leading to PGAM5 activation, which triggers FUNDC1-mediated mitochondrial autophagy (Wu et al., 2020). At the same time, targeting mitochondrial iron metabolism can also induce mitochondrial autophagy to inhibit tumor growth and metastasis, thus inhibit proliferation and migration and induce cell death (Sandoval-Acuña et al., 2021).

### 4.3 FUNDC1 is involved in maintaining protein homeostasis in mitochondrial matrix

Protein homeostasis is that cells ensure the correct folding and function of proteome through a series of quality control mechanisms, and at the same time degrade misfolded or unnecessary proteins in time to maintain cell function and prevent diseases. When the protein in the cell is damaged or misfolded proteins continue to accumulate in the cell, if not cleared in time, it will lead to protein homeostasis imbalance and may produce protein toxicity. FUNDC1 is a protein located in the outer membrane of mitochondria, which is very important for maintaining protein homeostasis in mitochondrial matrix. By interacting with the molecular chaperone protein HSC70 in the cytoplasm, FUNDC1 promotes the mitochondrial translocation of unfolded cytoplasmic proteins and transports them to the mitochondrial matrix (Li et al., 2019a). This process involves the action of LONP1 enzymes and the formation of non-aggregate protein aggregates (MAPAs) when proteasome activity is inhibited. Studies have shown that LONP1 and mtHSP70 with inherent chaperone-like activity can stabilize the folding intermediate of OXA1L and promote mitochondrial protein folding (Shin et al., 2021). However, excessive accumulation of unfolded proteins in mitochondria can damage the integrity of mitochondria, which may activate AMPK and lead to cell senescence (Li et al., 2019b). In order to maintain cell homeostasis and function, FUNDC1-mediated mitochondrial autophagy helps to clear damaged proteins that cooperate with ubiquitin, especially under stress conditions (Kocaturk et al., 2022).

### 4.4 FUNDC1 mediates crosstalk between mitochondria and endoplasmic reticulum

Crosstalk between mitochondrial dysfunction and endoplasmic reticulum stress promotes mitochondrial phagocytosis (Dlamini et al., 2021). In cell biology, FUNDC1 is a key protein (Liu et al., 2021a) that regulates the communication between mitochondrial autophagy and endoplasmic reticulum in mitochondrial quality control. Mitochondrial autophagy is realized by calcium-dependent FUNDC1 phosphorylation at the endoplasmic reticulum-mitochondrial interface (Ponneri Babuharisankar et al., 2023). This communication occurs in a specific region called mitochondrial associated endoplasmic reticulum (MAMs) and is essential for maintaining intracellular calcium homeostasis and lipid metabolism (Bai et al., 2023). FUNDC1 promotes the stability of MAMs and participates in calcium ion transport (Lv et al., 2022) by interacting with inositol 1mine4 receptor 5-trisphosphate receptor (IP3R). At the same time, the calcium signal crosstalk between endoplasmic reticulum and mitochondria can also provide a strategy for the development of new drugs for kidney disease (Ge et al., 2024). Specifically, calcium ions are transported from endoplasmic reticulum to mitochondria through IP3R-glucose-regulated protein 75 (GRP75)-voltage-dependent anion channel 1 (VDAC1) complex to restore mitochondrial dynamic balance and reduce neuronal apoptosis (Xu A. et al., 2024). The IP3R-GRP75-VDAC1 pathway will lead to calcium overload (Gao et al., 2024). YuanM and his colleague (Yuan et al., 2022) have found that conditional knockout of GRP75 in mouse model results in impaired calcium transport from endoplasmic reticulum to mitochondria, thereby reducing mitochondrial oxidative stress and calcium overload. The interaction between FUNDC1 and IP3R3 contributes to the stability of MAMs. FUNDC1 can control mitochondrial integrity and cardiac function in obesity in an IP3R3-dependent manner, and further maintain mitochondrial calcium homeostasis (Ren et al., 2020) by interacting with FBXL2, the receptor subunit of SCF (SKP1/cullin/F-box protein) ubiquitin ligase complex.

### 4.5 FUNDC1 mediates crosstalk between mitochondria and lysosomes

**FIGURE 4:**
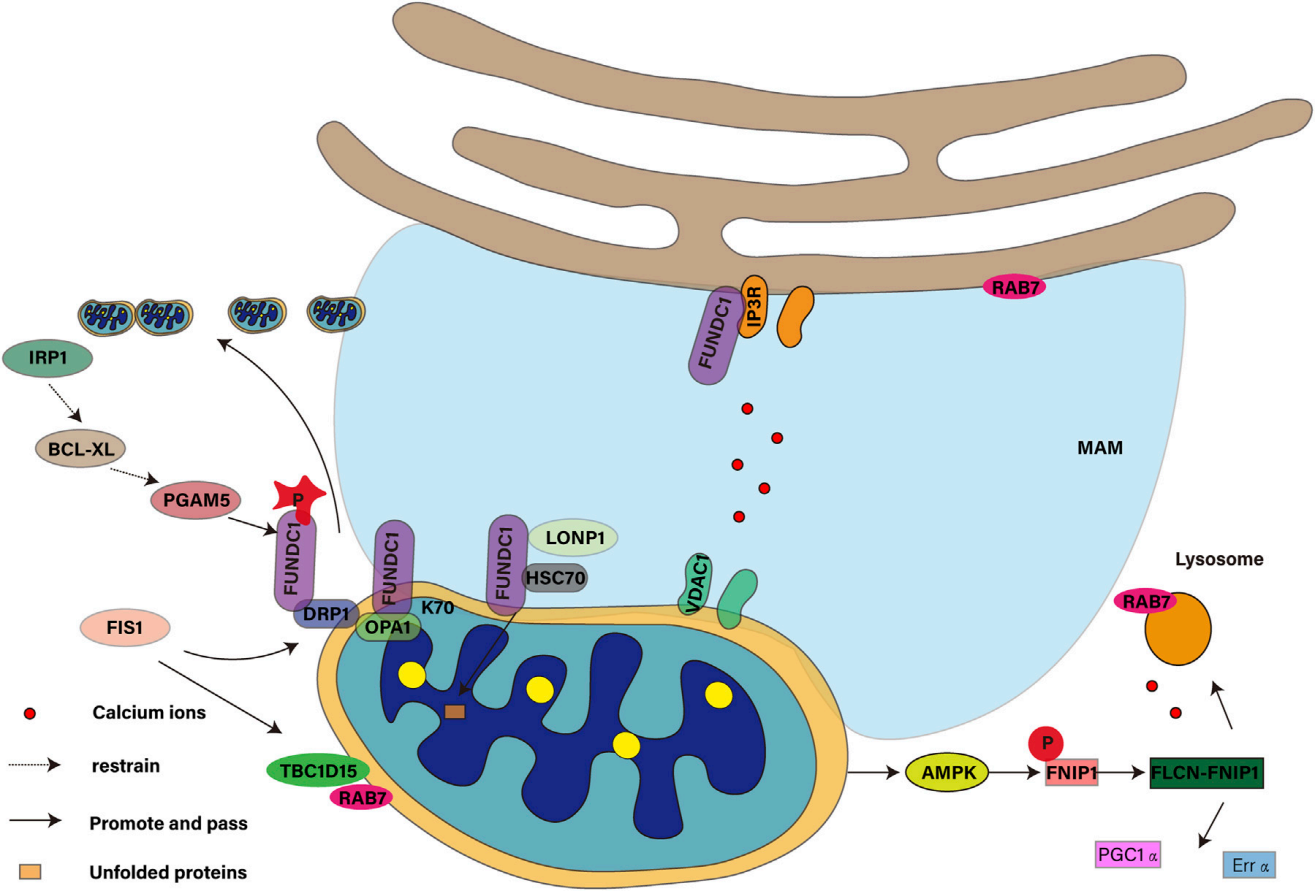
Role of the mitochondrial autophagy receptor FUNDC1 in mitochondrial events.

The interaction between mitochondria and lysosomes is essential for energy metabolism, calcium homeostasis and autophagy (Ureshino et al., 2019). Mitochondrial-lysosome contact is a key process in the degradation of damaged mitochondria by isolating damaged mitochondria into autophagosomes and then degrading them in lysosomes to remove dysfunctional mitochondria and maintain cell health (Peng et al., 2020). When AMPK is activated by mitochondrial damage, it phosphorylates FNIP1, initiates FLCN-FNIP1 complex and induces nuclear translocation of TFEB, which not only promotes the expression of PGC1a and Erra mRNAs (Malik et al., 2023), but also activates TFEB through lysosomal calcium release, which leads to calcineurin activation and mitochondrial autophagy (Oh et al., 2023). As the receptor of mitochondrial autophagy, FUNDC1 connects mitochondrial autophagy with biogenesis through PGC-1 α/NRF1 cascade regulation, and contributes to adaptive thermogenesis, so that cells can cope with mitochondrial damage and adjust metabolic state, thus maintaining mitochondrial homeostasis (Liu et al., 2021b). Rab7 is mainly distributed on lysosome, endoplasmic reticulum and mitochondrial membrane, which is responsible for membrane transport and regulates the maintenance or dissociation of mitochondrial-lysosome contact (Wong et al., 2018) through its activation state. As the GAP of RabGTP enzyme, TBC1D15 can mediate lysosome regeneration (Bhattacharya et al., 2023) and regulate mitochondrial fission by recruiting lysosomes from FIS1 to promote the transformation of Rab7-GTP to Rab7-GDP, thus relieving the contact between mitochondria and lysosomes (Wu et al., 2019). Studies have shown that TBC1D15/RAB7-regulated mitochondrial-lysosome interaction has a protective effect on heart injury induced by acute myocardial infarction (Yu et al., 2020). FIS1 can mediate TBC1D15 and DRP1 recruitment to promote mitochondrial fission (Ihenacho et al., 2023). Mid51/Fis1 mitochondrial oligomer complex can drive lysosome unbinding (Wong et al., 2022). FUNDC1 can mediate the overexpression of DRP1 receptors in MiD51 and Fis1, which are responsible for their mitochondrial recruitment, and promote mitochondrial autophagy (Roperto et al., 2019) in urothelial cells. Although FUNDC1 theoretically plays a key role in maintaining mitochondrial contact with lysosome and regulating mitochondrial fission and autophagy by regulating the expression of related proteins, there is no direct evidence that FUNDC1/MiD51/FIS1/TBC1D15/Rab7 pathway is involved in the regulation of mitochondrial-lysosome contact. Therefore, future studies need to provide more experimental data to support the effectiveness of this pathway and to verify the authenticity of FUNDC1-mediated interaction between mitochondria and lysosomes. As shown in Figure 4.

## 5 The role of mitochondrial autophagy receptor FUNDC1 in renal diseases

As an important excretory organ of the human body, the health status of the kidney is very important for the balance of the whole physiological system. The role of FUNDC1 in maintaining mitochondrial homeostasis and regulating mitochondrial autophagy has attracted more and more attention in recent years. In the context of kidney disease, FUNDC1-mediated mitochondrial autophagy may play a key role in cell response to injury and recovery. We will discuss the role of FUNDC1 in acute kidney injury (AKI), cardiorenal syndrome (CRS), diabetic nephropathy (DN), chronic kidney disease (CKD), renal fibrosis (RF) and renal anemia, aiming to provide new ideas and strategies for the treatment of kidney diseases.

### 5.1 AKI

AKI is a clinical syndrome with rapid decline of renal function caused by many factors, and renal ischemia-reperfusion injury (IRI) is the main cause (Li et al., 2024). In addition, patients with rhabdomyolysis and hemolysis may develop AKI (Mai et al., 2019; Goto et al., 2024). The occurrence of AKI is closely related to mitochondrial dysfunction, and the activation of mitochondrial autophagy is crucial to resist IRI (Su et al., 2023). The occurrence of AKI is closely related to mitochondrial dysfunction, and the activation of mitochondrial autophagy is very important to resist IRI. Studies have shown that regulating mitochondrial autophagy during IRI-AKI helps to maintain mitochondrial homeostasis and protect renal function (Lin et al., 2022). ZhangW and his colleague (Zhang et al., 2024) found that mitochondrial autophagy mediated by hypoxia inducible factor-1 α (HIF-1 α)/FUNDC1 signal transduction in renal tubular cells contributes to the prevention of renal IRI. LW6, a selective inhibitor of HIF-1 α, could reduce mitochondrial autophagy induced by hypoxia/reoxygenation, but increase apoptosis and ROS production. Hash R treatment could increase the expression of FUNDC1 protein, while the overexpression of FUNDC1 could reverse the effects of LW6 on the expression of LC3BII and voltage-dependent anion channels, block the cellular effect inhibited by HIF-1 α, and reduce apoptosis and ROS production. It is suggested that FUNDC1 can enhance the tolerance of cells to Hmax R condition by inducing mitochondrial autophagy. Studies by Zhang et al. (2022) have shown that renal ischemic preconditioning (IPC) reduces renal IRI, inflammation and macrophage infiltration in acute kidney by enhancing the autophagy activity of proximal renal tubular cells. Mitochondrial fission is an early molecular event in AKI, and its quality control, ROS oxidative stress and mitochondrial apoptosis are all strictly controlled. FUNDC1-induced mitochondrial autophagy may carry out IPC-mediated renal protection (Nesovic Ostojic et al., 2024) by weakening mitochondrial fission. IPC mediates mitochondrial autophagy by activating FUNDC1, which alleviates IRI-induced inflammation and renal function decline. Studies by Wang et al. (2020) have shown that IPC activates FUNDC1-mediated mitochondrial autophagy through ULK1 plays an important role in renal protection. Specific knockout of FUNDC1 in proximal renal tubules eliminates the protective effect of IPC on kidney, because IPC needs to degrade Drp1 located in mitochondria through FUNDC1-activated mitochondrial autophagy and inhibit IRI-activated mitochondrial division. Knockout of Drp1 can reverse the mitochondrial damage caused by FUNDC1 deletion and the ineffective response of IPC. In wild-type mice, Drp1 proximal tubule specific deletion can maintain the normal structure of mitochondria in damaged kidney, reduce oxidative stress, inflammation, programmed cell death and renal injury, and promote renal tubular epithelial repair (Perry et al., 2018). Therefore, targeted regulation of IPC-ULK1-FUNDC1-Drp1 axis has potential in clinical management of AKI.

### 5.2 CRS

CRS describes the interaction between the heart and the kidney, in which the failure of one organ affects the state of the other, which is of concern because of its high morbidity and mortality (Gallo et al., 2023). CRS can be divided into five types to reflect different initiation factors and pathological processes (He et al., 2021). Especially CRS-3 (Neres-Santos et al., 2021) and CRS-4 (Amador-Martínez et al., 2023) are closely related to the regulation of mitochondrial autophagy. The interdependence between the heart and the kidney is reflected in the dependence of the heart on the fluid regulation of the kidney and the dependence of the kidney on the blood flow and pressure produced by the heart (Johns, 2024). It is worth noting that hemodynamic changes are key drivers of CRS (Obi et al., 2016), such as increased central venous pressure and decreased cardiac output, which can lead to renal hypoperfusion and renal parenchyma hypoxia. In this low blood flow state, autophagy is activated as a cellular adaptation mechanism to maintain renal blood flow and slow down the development of CRS. FUNDC1 is a key regulator of mitochondrial autophagy, and its activation is usually related to hypoxia. Mitochondrial dysfunction plays a central role in heart failure (HF) and CKD. By activating mitochondrial autophagy, improving mitochondrial biology and maintaining mitochondrial homeostasis is helpful to break the vicious circle between HF and AKI/CKD (Shi et al., 2022). Cai et al. (2022b) believe that mitochondrial dysfunction is the key pathological mechanism of CRS-3 and that stimulating FUNDC1-dependent mitochondrial mass monitoring can improve mitochondrial function and cardiac function during CRS-3. Studies by WangJ and his team have shown that (Wang J. et al., 2022), Baxinhibitor-1 (BI-1) overexpression promotes myocardial mitochondrial autophagy and unfolded protein response, reduces mitochondrial oxidative stress, improves mitochondrial energy metabolism, and protects against heart damage caused by CRS-3. It was found that after CRS-3, Fundc1 and mt-Keima decreased, and mitochondrial autophagy was inhibited. However, BI-1 overexpression mice showed increased mitochondrial autophagy, and Fundc1 or Atf6 silencing weakened the protective effect of BI-1 on cardiomyocytes. Other studies have pointed out (Shen et al., 2023), Dapagliflozin (DAPA) can protect heart and kidney from CRS-4-associated cardiomyopathy by activating pyruvate kinase isoenzyme M2 (PKM2)/protein phosphatase 1 (PP1)/FUNDC1 mitochondrial autophagy. DAPA restored FUNDC1-dependent mitochondrial autophagy through PKM2-dependent pathway, while knockout of FUNDC1,DAPA could not protect myocardium and mitochondria. Although the role of FUNDC1 in CRS-3 and CRS-4 has been confirmed, its potential role in other types of CRS remains a mystery. CRS-1 and CRS-2 are mainly involved in the rapid deterioration of renal function after acute cardiac events, while CRS-5 is caused by both heart and kidney damage caused by systemic diseases. Future research needs to explore the role of FUNDC1 in these types of CRS and whether it can provide protective effects similar to those observed in CRS-3 and CRS-4.

### 5.3 DN

DN is one of the most common microvascular complications of diabetes, and it is also the main cause of end-stage renal disease, which is characterized by changes in renal structure and function (Chen J. et al., 2024). 30%–40% of patients with both type 1 and type 2 diabetes are likely to develop kidney damage (Li et al., 2021). In the state of diabetes, the glucose metabolism of the kidney is significantly enhanced, and 60% of the endogenous glucose released after a meal is metabolized in the kidney, which increases the glucose load of the kidney (Alsahli and Gerich, 2017). Podocyte is a key component of glomerular filtration barrier, and its health status is very important to prevent the development of DN (Wang S. et al., 2022). The disorder of mitochondrial dynamics is an important mechanism of podocyte injury in DN, in which the signal molecule FUNDC1 plays an important role in regulating mitochondrial homeostasis. In the normal state, FUNDC1 binds to the OPA1 of the mitochondrial inner membrane, which makes the mitochondria tend to fuse; in the stress state, FUNDC1 and OPA1 dissociate and recruit the DRP1 in the cytoplasm to bind to the mitochondria, thus promoting mitochondrial division. FUNDC1 can restore the homeostasis of mitochondria by mediating mitochondrial autophagy, regulating the level of mitochondrial fission and inhibiting the indexes related to mitochondrial fusion. In high glucose environment, the activation of Src was positively correlated with renal dysfunction. ZhengT and colleagues (Zheng et al., 2022) found that Src activation leads to FUNDC1 phosphorylation, inhibition of mitochondrial autophagy, podocyte damage and DN progression, inhibition of Src activity can protect podocytes from mitochondrial damage in high glucose environment, but FUNDC1 silencing eliminates the protective effect of inhibiting Src activity. PAA activates mitochondrial autophagy by down-regulating FUNDC1, increasing the levels of LC3 and ATG5, and reducing the level of p62. The downregulation of FUNDC1 further enhanced the protective effect of PAA on MPC5 cells after HG treatment, indicating that downregulation of FUNDC1-induced mitochondrial autophagy can reduce DN podocyte injury (Wu et al., 2023). WeiX and his colleague (Wei et al., 2020) have found that capsaicin reduces Fundc1 transcription by activating TRPV1 and AMPK, thereby alleviating podocyte mitochondrial dysfunction caused by hyperglycemia and improving DN. At the same time, inhibition of AMPK or overexpression of Fundc1 will prevent this protective effect.

### 5.4 CKD

CKD leads to irreversible decline of renal function with the passage of time. The core problems of CKD are decreased glomerular filtration rate and renal structural fibrosis (Yang et al., 2024). Abnormal mitochondrial autophagy is a common pathogenesis of CKD. FUNDC1-mediated mitochondrial autophagy plays a role in this process. By clearing the damaged mitochondria, it helps to reduce RF, protect renal function and delay the progress of CKD. In the early stage of CKD, when the renal oxygen supply is insufficient, the mitochondria produce ROS, which leads to mitochondrial dysfunction. FUNDC1 activation guides the damaged mitochondria to autophagy to prevent cell death and RF caused by mitochondrial dysfunction. However, in the late stage of CKD, persistent renal injury and inflammation inhibit the expression of FUNDC1, reduce mitochondrial autophagy, lead to the accumulation of damaged mitochondria, aggravate fibrosis and decrease renal function. Therefore, regulating the expression and activity of FUNDC1 and restoring mitochondrial autophagy is a potential strategy for the treatment of CKD. Ma et al. (2021) found that inhibition of mitochondrial autophagy activation can lead to renal tubular necrosis and RF in CKD, while mediating mitochondrial autophagy can effectively inhibit cisplatin-induced CKD inflammation and RF. In a study by WeiX and his team (Wei et al., 2023), they found the preventive effect of magnolol on chronic kidney disease. This effect is realized by mitochondrial autophagy and AMPK pathway mediated by BNIP3/NIX and FUNDC1. Magnolol can inhibit the expression of BNIP3, NIX and FUNDC1, thus reduce the phenomenon of mitochondrial autophagy in CKD rats, and play a protective role in the kidney of CKD rats.

### 5.5 RF

RF is the main pathological feature of CKD and end-stage renal disease, involving inflammation, oxidative stress, epithelial-mesenchymal transition (EMT), and excessive deposition of extracellular matrix (ECM). In healthy kidneys, mitochondria maintain their morphology and function through continuous fusion and fission (Sun J. et al., 2024). In RF, this dynamic balance is disrupted, resulting in increased mitochondrial fission and abnormal autophagy. Chen H and colleagues (Chen H. et al., 2024) found that impaired mitochondrial autophagy aggravates RF, and the Mfn2-MAMs-FUNDC1 pathway plays an important role in reversing RF. Vitamin D receptor can affect the integrity of MAMs by interacting with Mfn2, thereby regulating the function of FUNDC1, restoring mitochondrial autophagy, reducing mitochondrial fission, reducing mitochondrial ROS production, and increasing mitochondrial membrane potential and ATP production, thereby protecting RF. The role of mitochondrial autophagy in kidney disease is complex and dual-sided. It can play a protective or harmful role in different pathological states. In some cases, the activation of autophagy can remove damaged organelles and proteins and maintain the stability of the intracellular environment, thus having a renal protective effect. As mentioned above, restoring mitochondrial autophagy can alleviate renal fibrosis. However, under conditions of persistent stress or injury, excessive or persistent autophagy may aggravate cell damage and RF. 3-Methyladenine (3-MA) is a specific autophagy inhibitor that can inhibit autophagy by blocking the formation of autophagosomes and preventing the nucleation stage of autophagy. Studies have found that 3-MA can not only significantly reduce the number of autophagic vacuoles in the kidneys of diseased rats, inhibit mitochondrial fission, reduce the expression of Drp-1, Cofilin and F-actin, and alleviate cell apoptosis; but also reduce the arrest of the G2/M phase of the cell cycle in the kidneys by inhibiting autophagy, inhibit EMT, and reduce the deposition of ECM proteins, thereby alleviating RF (Shi et al., 2020). Therefore, therapeutic strategies targeting autophagy need to be customized according to specific pathological conditions and disease stages.

### 5.6 Renal anemia

**FIGURE 5:**
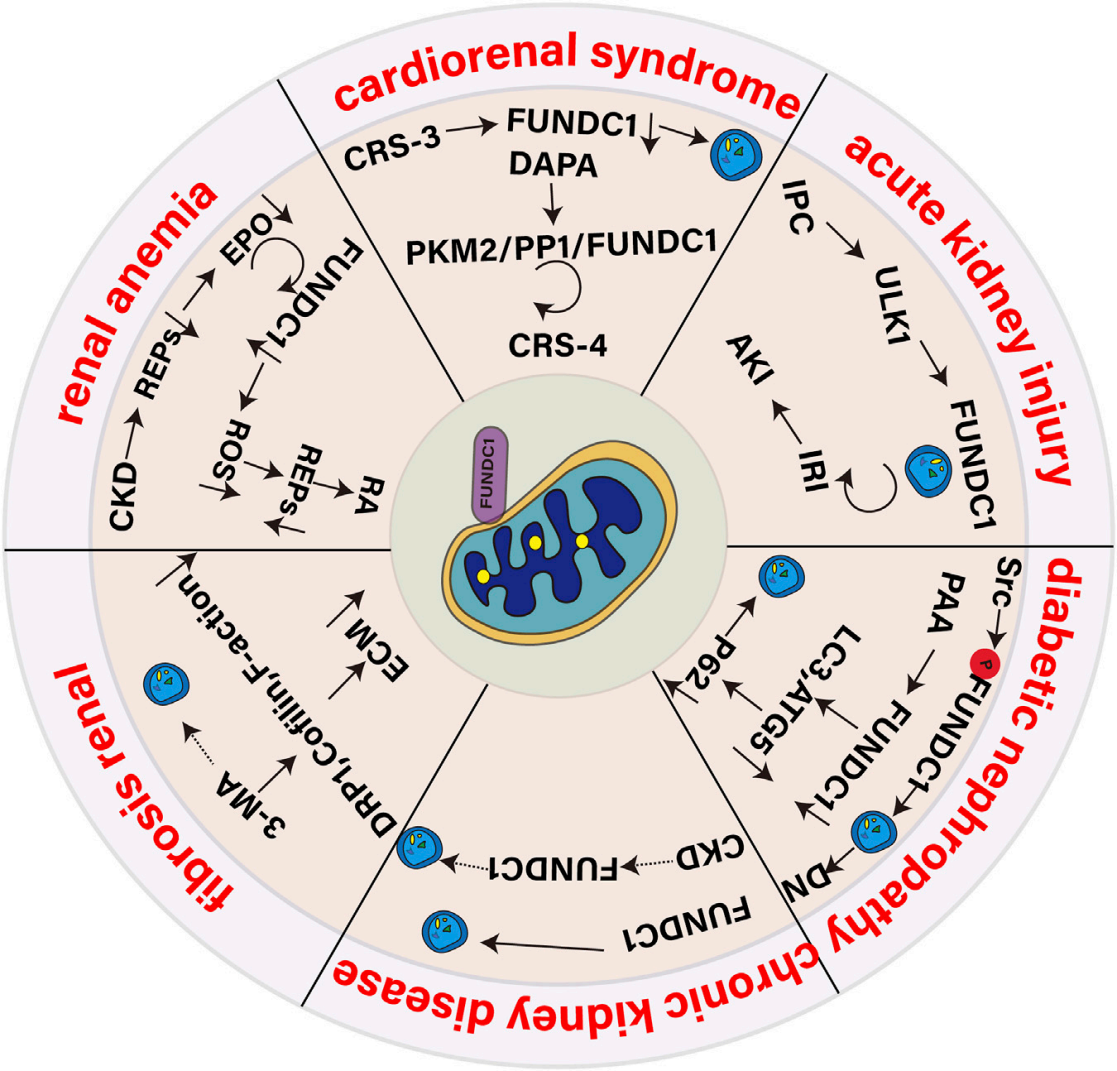
Role of the mitophagy receptor FUNDC1 in kidney disease.

Renal anemia is one of the common complications of CKD, and its main cause is the reduction of erythropoietin (EPO) produced by the kidney (Hitomi et al., 2017). EPO is a key cytokine that drives erythropoiesis in the bone marrow, and the adult kidney is its main production site (Tomc and Debeljak, 2021). FUNDC1, as a mitochondrial autophagy receptor, is essential for EPO-driven erythropoiesis under stress conditions. In the CKD state, renal EPO-producing cells (REPs) are damaged, resulting in reduced synthesis and secretion of EPO (Schley and Hartner, 2022). FUNDC1 helps maintain mitochondrial homeostasis and function of REPs by promoting selective autophagy of damaged mitochondria. When FUNDC1 function is impaired, damaged mitochondria accumulate in REPs, leading to increased ROS levels, triggering an inflammatory response, which in turn affects the function of renal REPs and ultimately leads to renal anemia. In addition, impaired FUNDC1 function further promotes the transformation of REPs into myofibroblasts by upregulating the expression of proinflammatory cytokines. This transformation leads to a decrease in EPO production capacity, thereby exacerbating renal anemia. Geng G and colleagues (Geng et al., 2021) showed that the loss of the FUNDC1 gene exacerbated RF in cisplatin-induced renal anemia and unilateral ureteral obstruction models, a phenomenon attributed to the accumulation of damaged mitochondria, increased oxidative stress, and inflammatory responses. By enhancing FUNDC1-mediated mitochondrial autophagy, these damaged mitochondria can be cleared, oxidative stress and inflammation can be reduced, REPs in the kidney can be protected, and normal EPO production can be maintained, thus providing a potential therapeutic strategy for the treatment of renal anemia and RF. Although HIF (hypoxia-inducible factor) plays a key role in regulating EPO expression, especially under hypoxic conditions, HIF upregulates its expression by binding to the enhancer region of the EPO gene (Farsijani et al., 2016), current studies have not directly mentioned that FUNDC1-mediated processes involve HIF. FUNDC1 mainly regulates EPO production by affecting mitochondrial quality and autophagy, rather than directly through the HIF pathway. However, since both HIF and mitochondrial function are related to the cellular response to hypoxia, there may be an indirect connection between them, but further studies are needed to clarify their interaction. As shown in Figure 5.

## 6 Discuss

This article focuses on the role of the mitophagy receptor FUNDC1 in maintaining mitochondrial function and kidney disease. FUNDC1 regulates mitochondrial autophagy through phosphorylation and dephosphorylation, which is essential for cell energy metabolism and programmed death. The phosphorylation status of FUNDC1 is regulated by multiple kinases and phosphatases, including ULK1, Src, CK2, PGAM5 and BCL2L1. In mitochondrial events, the role of FUNDC1 is not limited to promoting mitochondrial autophagy. It also participates in mitochondrial fission, maintains the homeostasis of iron and proteins in mitochondrial matrix, and plays a role in crosstalk between mitochondria, endoplasmic reticulum and lysosomes. These functions indicate that FUNDC1 is the intersection of multiple signal pathways in cells, and its abnormal function may lead to a variety of cellular dysfunction. In the context of kidney disease, the imbalance of mitochondrial autophagy mediated by FUNDC1 is closely related to the development of AKI, CRS, DN, CKD, RF, and renal anemia. These findings suggest that FUNDC1 may be a potential therapeutic target and its regulation may help to restore mitochondrial function and improve the prognosis of renal disease.

However, we also found some limitations. First, the current understanding of the phosphorylation and dephosphorylation mechanisms of FUNDC1 protein is incomplete, and further reviews are needed to reveal its exact functions in various disease states. Second, the effects of FUNDC1 may be different in different types of kidney disease. Although we have explored the role of FUNDC1 in AKI, CRS, DN, CKD, and RF, given that the pathogenesis of kidney disease is closely related to mitochondrial autophagy, and there is a lack of clinical reports on other types of kidney diseases such as hyperuricemia nephropathy and lupus nephritis, this field needs more clinical data to support it. Finally, how to precisely regulate the activity of FUNDC1 and transform these research results into clinical treatment methods will be the key direction of future research.

In recent years, studies have found that “intestinal flora metabolism-FUNDC1-mediated mitochondrial autophagy” pathway may be a new way to improve inflammatory damage in renal disease. The imbalance of metabolic function of intestinal flora may lead to oxidative stress of mitochondria and increase of ROS (Sun Y. et al., 2024). These factors may be the key to the activation of NLRP3 inflammatory bodies (Shi et al., 2024; Xu C. et al., 2024) and the main pathological mechanism of inflammatory damage in renal disease (Chang et al., 2024). The phosphorylation of FUNDC1 may affect the activity of mitochondrial autophagy, which in turn affects the inflammatory response of kidney disease. Based on this, the authors propose the following hypothesis: in the process of renal disease, intracellular homeostasis and inflammatory damage are affected by intestinal flora regulation and FUNDC1-NLRP3-mediated mitochondrial homeostasis disorders. The dysfunction of FUNDC1, such as inhibition of mitochondrial autophagy and excessive activation of mitochondrial oxidative stress, may lead to a vicious circle of intracellular damage mechanisms, aggravate inflammatory response, and promote apoptosis in the mitochondrial pathway. By regulating the abundance of intestinal flora to activate FUNDC1-mediated mitochondrial autophagy and inhibit NLRP3-mediated inflammation, we can adjust the homeostasis of intracellular environment and treat renal disease. In short, FUNDC1 is a key mitochondrial autophagy regulator, and its role in maintaining mitochondrial function and kidney disease is worthy of further exploration. Future research will help to better understand its role in diseases and provide a theoretical basis for the development of new treatment strategies.

## 7 Conclusion

Mitochondrial autophagy receptor FUNDC1 plays a key role in maintaining mitochondrial function and kidney disease. Through the dynamic regulation of phosphorylation and dephosphorylation, FUNDC1 participates in the activation and inhibition of mitochondrial autophagy, thus affecting the fate of cells. Its role in the development of renal disease suggests its potential as a potential therapeutic target. Future research needs to further explore the molecular mechanism of FUNDC1 and its application in the treatment of renal diseases.
